# Biomarcadores Potenciais na Fibrose Miocárdica: Uma Análise Bioinformática

**DOI:** 10.36660/abc.20230674

**Published:** 2024-11-22

**Authors:** Wang Cheng-Mei, Gang Luo, Ping Liu, Wei Ren, Sijin Yang

**Affiliations:** 1 Beibei Traditional Chinese Medicine Hospital Chongqing China Beibei Traditional Chinese Medicine Hospital, Chongqing - China; 2 The Affiliated Hospital of Traditional Chinese Medicine Southwest Medical University Luzhou Sichuan China The Affiliated Hospital of Traditional Chinese Medicine of Southwest Medical University, Luzhou, Sichuan – China

**Keywords:** Fibrose Endomiocárdica, Biomarcadores, MicroRNAs

## Abstract

**Fundamento:**

A fibrose miocárdica (FM) ocorre durante o início e a progressão da doença cardiovascular, e o diagnóstico precoce da FM é benéfico para melhorar a função cardíaca, mas há uma falta de pesquisa sobre biomarcadores precoces da FM.

**Objetivos:**

Utilizando técnicas de bioinformática, identificamos potenciais biomarcadores para FM.

**Métodos:**

Os conjuntos de dados relacionados à FM foram obtidos do banco de dados Gene Expression Omnibus (GEO). Após o processamento dos dados, genes diferencialmente expressos foram rastreados. Genes diferencialmente expressos foram enriquecidos e, subsequentemente, interação proteína-proteína (PPI) foi realizado para analisar os genes diferenciais. Os miRNAs associados e fatores de transcrição foram previstos para esses genes centrais. Finalmente, a validação ROC foi realizada nos genes centrais para determinar sua especificidade e sensibilidade como potenciais biomarcadores. O nível de significância adotado foi de 5% (p < 0,05).

**Resultados:**

Um total de 91 genes diferencialmente expressos foram identificados, e a análise PPI produziu 31 genes centrais. A análise de enriquecimento mostrou que apoptose, colágeno, matriz extracelular, adesão celular e inflamação estavam envolvidos na FM. Cento e quarenta e dois miRNAs potenciais foram identificados. Os fatores de transcrição JUN, NF-κB1, SP1, RELA, SRF e STAT3 foram enriquecidos na maioria dos alvos principais. Por fim, IL11, GADD45B, GDF5, NOX4, IGFBP3, ACTC1, MYOZ2 e ITGB8 tiveram maior precisão diagnóstica e sensibilidade na predição de FM com base na análise da curva ROC.

**Conclusão:**

Oito genes, IL11, GADD45B, GDF5, NOX4, IGFBP3, ACTC1, MYOZ2 e ITGB8, podem servir como biomarcadores candidatos para FM. Processos como apoptose celular, síntese de proteína de colágeno, formação de matriz extracelular, adesão celular e inflamação estão implicados no desenvolvimento da FM.

## Graphical abstract

**Figura Central:**
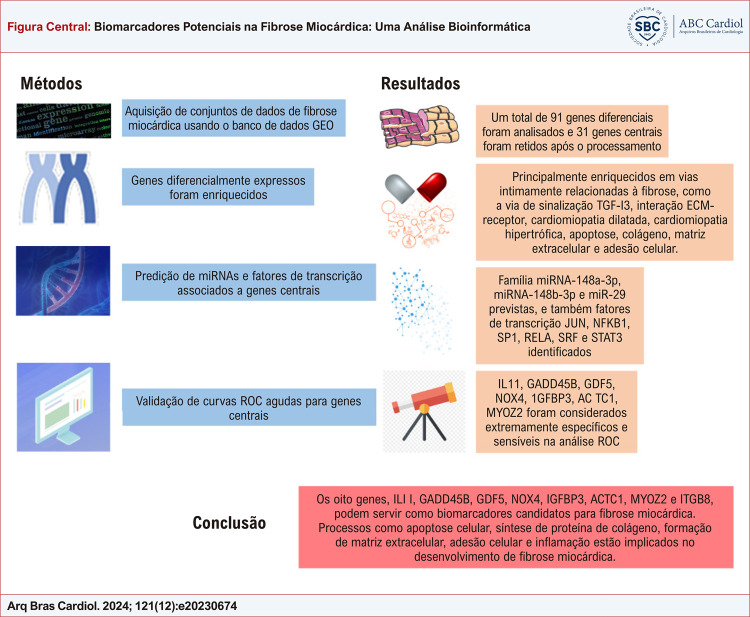
: Biomarcadores Potenciais na Fibrose Miocárdica: Uma Análise Bioinformática

## Introdução

As doenças cardiovasculares são comuns e a sua taxa de mortalidade ultrapassa a de outras doenças sistémicas.^1^ Eles ceifam milhões de vidas anualmente, constituindo aproximadamente 25% das mortes em todo o mundo.^2^ A fibrose miocárdica (FM) desempenha um papel fundamental no início e na progressão de condições como fibrilação atrial, doença arterial coronária, infarto do miocárdio, cardiomiopatia dilatada e cardiomiopatia hipertrófica.^3-6^ A FM é causada pela deposição anormal de tecidos fibrosos no miocárdio. É caracterizada por um acúmulo excessivo da matriz extracelular (MEC) e um aumento significativo de colágeno, levando à rigidez cardíaca e ao declínio da função cardíaca, seguido por remodelação elétrica, arritmias e insuficiência cardíaca.^7^

Do ponto de vista fisiopatológico, a FM está intrinsecamente ligada aos mecanismos reparadores após dano cardíaco. Cardiomiócitos são células não regenerativas. Quando essas células são danificadas ou morrem, as células circundantes liberam uma série de moléculas de sinalização, como mediadores neuro-humorais, citocinas e fatores de crescimento, que induzem a ativação dos fibroblastos para entrar na área danificada e produzir grandes quantidades de colágeno.^8^ O colágeno é um componente primário da estrutura do tecido e seu acúmulo excessivo pode levar à deposição anormal da matriz extracelular, reduzindo assim a complacência cardíaca e prejudicando sua função.^9^ À medida que a fibrose progride, as funções sistólica e diastólica do coração ficam ainda mais comprometidas, resultando em insuficiência cardíaca e afetando significativamente a qualidade de vida dos pacientes.

Muitas doenças cardiovasculares podem se manifestar como FM em estágio inicial.^10^ Com o envelhecimento da população global e o aumento de doenças relacionadas ao estilo de vida, como fibrilação atrial, hipertensão, doença cardíaca coronária e diabetes, a incidência de FM está aumentando constantemente, representando uma grave ameaça à saúde humana. Lamentavelmente, os tratamentos atuais se concentram principalmente em retardar a progressão da fibrose, mas não podem revertê-la efetivamente.^11^ Isso ocorre porque os cardiomiócitos não conseguem substituir adequadamente as células mortas, e o processo de fibrose envolve células e vias de sinalização extremamente complexas. Portanto, com os avanços tecnológicos em chips de genes, técnicas de sequenciamento e sequenciamento de genoma completo, a identificação precoce da FM e a busca por biomarcadores altamente sensíveis e específicos para elaborar novas estratégias terapêuticas para reverter essa condição se tornam primordiais.

Originário de uma perspectiva de bioinformática, o estudo identificou e analisou genes hub diferencialmente expressos na FM, investigando suas perspectivas como biomarcadores. Os processos centrais deste estudo podem ser visualizados na Figura Central.

## Métodos

### Obtendo um GeneChip

O *Gene Expression Omnibus*^12^ (GEO) foi usado para rastrear três conjuntos de dados de microarray relacionados à FM e seus arquivos de anotação genética, especificamente GSE 123018/GPL11154, GSE152250/GPL20301 e GSE225336/GPL24676. Os dados brutos do GSE123018 continham dados de quatro indivíduos; GSE152250 e GSE225336 usaram dados de três indivíduos. Todos os três conjuntos de dados continham cardiomiócitos induzidos por 24 horas usando TGFβ1, então escolhemos o conjunto de dados processado em 24 horas e, para tornar os dados menos diferenciados, selecionamos aleatoriamente os dados celulares dos três pacientes no GSE123018. Todos esses dados são de código aberto e, portanto, não exigem revisão por um comitê de ética.

### Triagem de genes expressos diferencialmente

Arquivos relacionados ao chip genético foram obtidos e baixados do banco de dados GEO. Esta pesquisa utilizou conjuntos de dados de três origens distintas, levando a variações no manuseio laboratorial de cada chip genético. Consequentemente, a remoção de efeitos de lote e a normalização de dados se tornaram primordiais.^13^ O pacote sva em R (Versão 4.2.1) foi inicialmente aplicado para estimativa de efeito de lote, seguido pelo emprego da função de combate para sua mitigação. Modelos lineares e métodos bayesianos no pacote *limma* foram usados para avaliar os níveis de expressão gênica sob diferentes condições experimentais, corrigir as estimativas de variância de genes individuais e normalizar as matrizes gênicas usando a função *normalize BetweenArrays*, respectivamente. Para selecionar genes com expressão diferencial significativa, definimos um limite de |log2-fold change| valor maior que 2 e valor p menor que 0,05 nos critérios de seleção. Técnicas de visualização foram implementadas com os pacotes *ggplot2* e *pheatmap*.

### Análise de interações proteína-proteína

A análise da interação proteína-proteína (PPI)^14^ oferece insights sobre com quais outras proteínas uma proteína específica interage dentro de um organismo. Isso ajuda a caracterizar melhor as funções dessas proteínas e pode lançar luz sobre o início e a progressão da doença. Genes diferencialmente expressos (GDE) foram inseridos no banco de dados STRING (https://stringdb.org),^15^ que é uma ferramenta de análise online que inclui pontuação de integração de dados, modelagem probabilística, teste hipergeométrico, análise de topologia de rede e correção de taxa de descoberta falsa (TDF), e o arquivo TSV da rede PPI foi exportado. Este arquivo foi posteriormente carregado no Cytoscape (versão 3.8.2)^16^ para visualização. O plugin CytoNCA calcula métricas de alvo como grau, proximidade e intermediação, permitindo a seleção de alvos principais. O plugin MCODE é então utilizado para identificar potenciais complexos biomoleculares.

### Análise de enriquecimento genético

Utilizando o Metascape (https://Metascape.org/),^17^ uma plataforma web para análise de enriquecimento funcional de grandes conjuntos de genes, Gene Ontology (GO) e Enciclopédia de Genes e Genomas de Kyoto (KEGG) análises de GDE e potenciais complexos biomoleculares foram realizadas usando testes hipergeométricos, método de correção de teste múltiplo do método Benjamini-Hochberg. Entradas com um valor p < 0,01 foram consideradas significativamente enriquecidas, e os 15 principais resultados da análise de enriquecimento GO e das vias KEGG foram selecionados.

### Análise de enriquecimento do conjunto de genes

Para melhorar de forma abrangente a compreensão das funções dos genes, o software GSEA (versão 4.3.2)^18^ foi empregado em todo o conjunto de dados para análise de enriquecimento funcional, abrangendo as vias GO, KEGG e análise Reactome. Para determinar o enriquecimento significativo, os critérios foram estabelecidos da seguinte forma: um escore de enriquecimento normalizado superior a 1,5, um valor de p nominal inferior a 0,05 e uma TDF inferior a 0,25.

### Aquisição de miRNAs críticos e fatores de transcrição

Os genes Hub foram analisados para potencial miRNA usando o software FunRich (versão 3.1.3),^19^ e a predição do fator de transcrição foi realizada usando TRRUST versão 2 (https://www.grnpedia.org/trrust/).^20^

### Validação do conjunto de genes com base na curva característica de operação do receptor (ROC)

Para validar a robustez dos genes hub identificados e evitar overfitting dos dados originais, é crucial conduzir a validação ROC^21^ em um conjunto de dados independente para avaliar os genes hub como potenciais biomarcadores. Utilizamos o conjunto de dados de microarray de genes GSE97358, que inclui 84 amostras normais e 84 amostras fibróticas. A análise e visualização ROC foram conduzidas no software R usando o pacote pROC.

## Resultados

### Análise de GDEs

**Figura 1:**
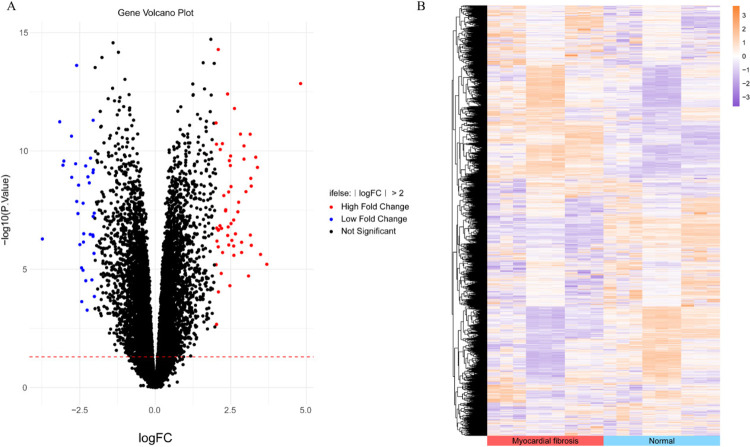
– A) Gráfico do vulcão genético. B) Mapa de calor genético.

Após o conjunto de dados original ter sido corrigido em lote e normalizado, uma análise diferencial foi conduzida usando o pacote limma. Por fim, 91 GDE foram identificados, com 56 genes significativamente regulados positivamente e 35 genes significativamente regulados negativamente. Mapas de calor de genes e gráficos de vulcão de GDE são ilustrados na Figura 1. Os parâmetros topológicos específicos dos GDE podem ser encontrados na Tabela S1.

### Análise PPI

**Figura 2:**
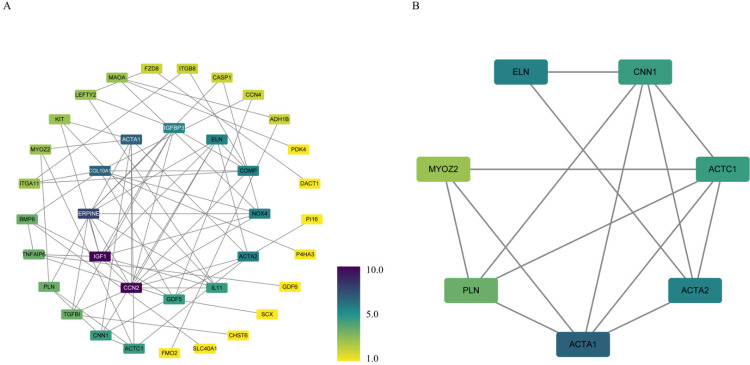
– A) Rede PPI. B) módulos biofuncionais.

Os 91 GDE foram carregados no site STRING para análise PPI. Os resultados da análise foram importados para o Cytoscape para investigação posterior. A Figura 2A descreve a rede consistindo em 37 nós e 71 arestas. Os graus de gene foram calculados usando CytoNCA e, ao classificar valores maiores que a mediana, 28 genes hub foram identificados. Os parâmetros topológicos específicos dos genes hub podem ser encontrados na Tabela S2. Enquanto isso, o plugin MCODE revelou um potencial módulo funcional de complexo de proteína (Figura 2B).

### Enriquecimento funcional

**Figura 3:**
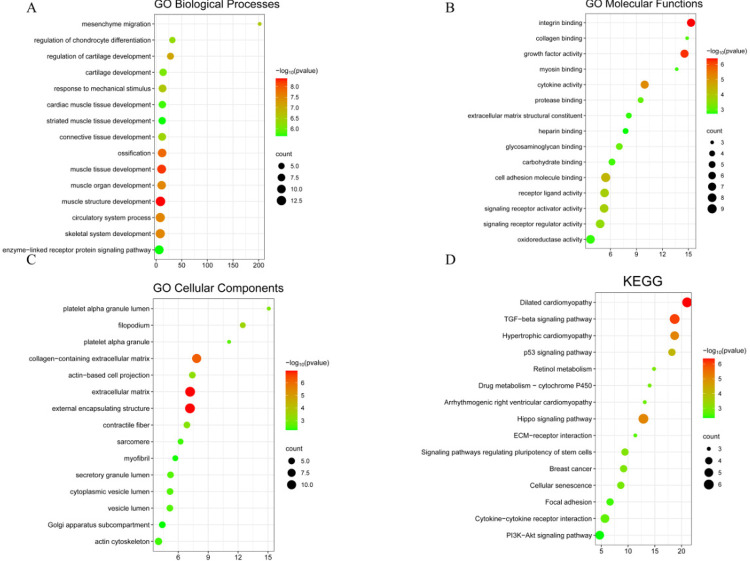
– A) Processos biológicos do GO. B) Função molecular do GO. C) Componentes celulares do GO. D) KEGG.

**Figura 4:**
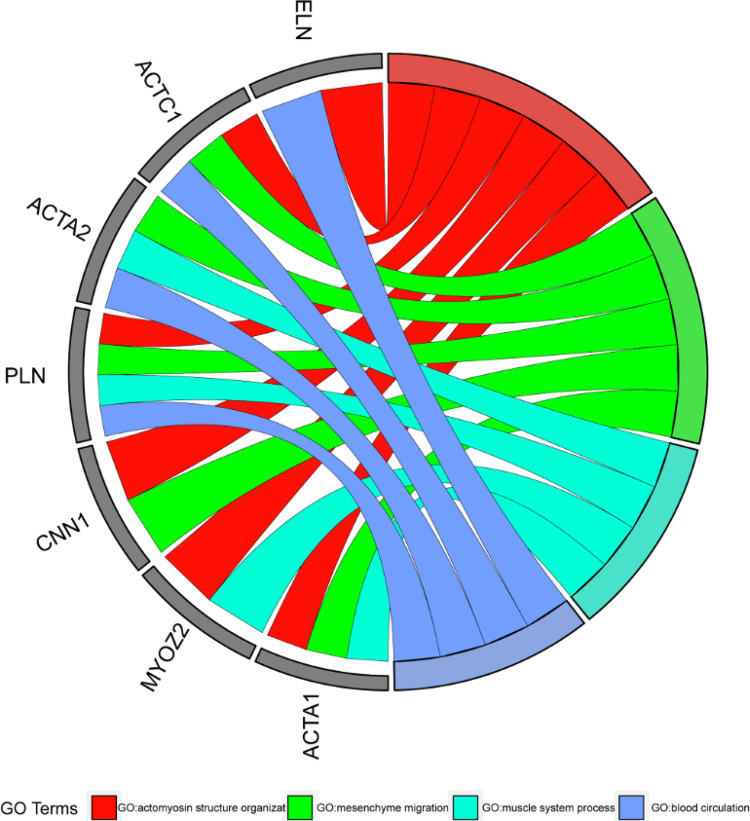
– Análise de enriquecimento do módulo biológico.

A análise da expressão gênica diferencial destacou que, como mostrado na Figura 3A, os genes participam de processos biológicos como migração mesenquimal, desenvolvimento do tecido conjuntivo, desenvolvimento do tecido miocárdico, resposta a estímulos mecânicos, processo do sistema circulatório e desenvolvimento do tecido muscular estriado. A Figura 3B demonstra o envolvimento em várias funções biológicas moleculares, como ligação da molécula de adesão celular, ligação da integrina, atividade do fator de crescimento, ligação do glicosaminoglicano, ligação do colágeno, constituintes estruturais da matriz extracelular e atividade da oxidorredutase. Os componentes celulares implicados incluem a MEC, estruturas encapsulantes externas, MEC contendo colágeno, lúmen do grânulo secretor, lúmen da vesícula citoplasmática e miofibrilas, como mostrado na Figura 3C. A análise da via KEGG indicou envolvimento em múltiplas vias de sinalização, como a via de sinalização do hipopótamo, interação citocina-receptor de citocina, cardiomiopatia hipertrófica, interação MEC-receptor, cardiomiopatia arritmogênica do ventrículo direito, senescência celular, fator de crescimento transformador-β(TGF-β)via de sinalização, Cardiomiopatia dilatada e via de sinalização p53, que podem ser observadas na Figura 3D. Todas as análises de enriquecimento com valores de p menores que 0,05 são mostradas na Tabela S3. A análise de enriquecimento do módulo biológico mostrou envolvimento na montagem da MEC e na migração mesenquimal, conforme mostrado na Figura 4.

### Resultados GSEA

**Figura 5:**
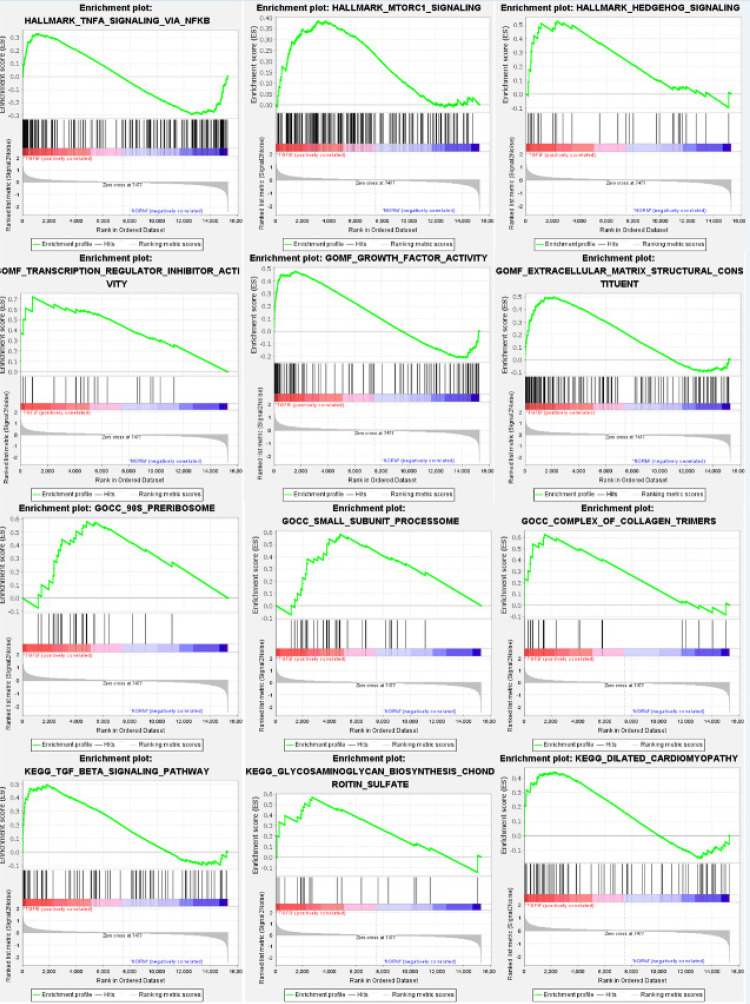
– GSEA.

Ao conduzir GSEA no conjunto de dados de genes totalmente ajustado, o Reactome revelou enriquecimentos primários em TGF-β, sinalização do fator de necrose tumoral Hallmark-α (TNF-α) via NF-κB, sinalização mTorc1, sinalização Hedgehog e resposta de proteína desdobrada. Os principais componentes celulares incluíram o processoma da subunidade, pré-ribossomo e trímeros complexos de colágeno. Em termos de funções biológicas, a participação do gene foi observada na atividade do inibidor do regulador de transcrição, constituinte estrutural da MEC e atividade do fator de crescimento. A análise da via KEGG mostrou envolvimento na via de sinalização TGF-β, biossíntese de glicosaminoglicanos, sulfato de condroitina e cardiomiopatia dilatada. Lamentavelmente, nos processos biológicos GO, nenhum conjunto de genes foi significativo em uma TDF abaixo de 25%, conforme ilustrado na Figura 5, e os parâmetros relevantes podem ser encontrados na Tabela S4.

### Predição de miRNAs e fatores de transcrição

**Figura 6:**
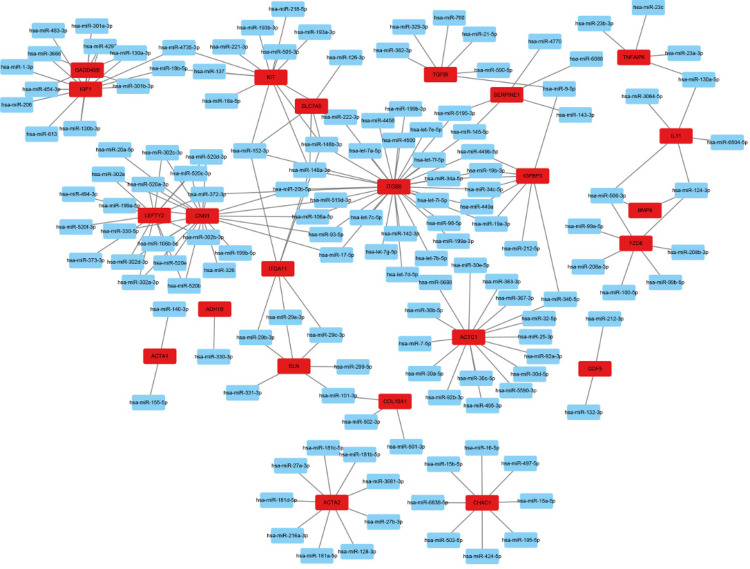
– miRNAs.

**Figura 7:**
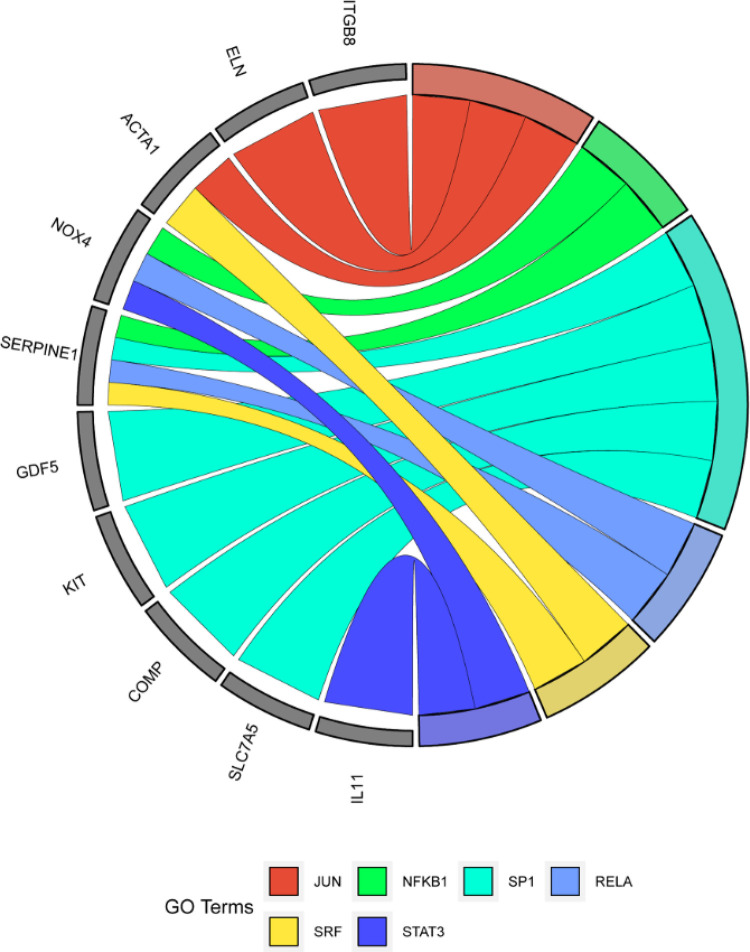
– Análise dos fatores de transcrição.

Utilizando FunRich para prever miRNAs, a análise de abundância reteve miRNA-148a-3p, miRNA-148b-3p, miR-152-3p, miR-372-3p, miR-506-3p, a família miR-130, a família miR-302, a família miR-29 e a família miR-520, conforme exibido na Figura 6. A previsão pós-análise de fatores de transcrição por meio da plataforma TRRUST revelou que o proto-oncogene Jun (JUN), NF-κB1, fator de transcrição Sp1(SP1), proto-oncogene RELA (RELA), fator de resposta sérica (SRF), e transdutor de sinal e ativador da transcrição 3 (STAT3) foram altamente enriquecidos entre a maioria dos alvos principais, conforme ilustrado na Figura 7.

### Os resultados da validação do ROC

Durante a análise ROC dos principais alvos, o estudo descobriu que as AUCs para os genes interleucina 11 (IL11), beta induzível por parada do crescimento e dano ao DNA (GADD45B), fator de diferenciação do crescimento 5 (GDF5), NADPH oxidase 4 (NOX4), proteína de ligação ao fator de crescimento semelhante à insulina 3 (IGFBP3), actina alfa do músculo cardíaco 1 (ACTC1), miozenina 2 (MYOZ2) e subunidade beta 8 da integrina (ITGB8) foram 0,964710884, 0,943239796, 0,936791383, 0,929563492, 0,92651644, 0,926232993, 0,91723356 e 0,906391723, respectivamente. Todas as AUCs foram maiores que 0,9; entre elas, IL11, GADD45B, NOX4, IGFBP3 e ACTC1 são reguladas positivamente na fibrose, enquanto GDF5, MYOZ2 e ITGB8 são reguladas negativamente na expressão, comprovando esses genes como potenciais marcadores biológicos para o diagnóstico de FM.

## Discussão

As doenças cardiovasculares, como principal causa de morte em todo o mundo, representam um fardo significativo para a saúde pública global.^1^ Condições cardiovasculares comuns, como doença cardíaca coronária, infarto do miocárdio, doença cardíaca hipertensiva, cardiomiopatia, fibrilação atrial e cardiomiopatia diabética, podem levar à FM, resultando em insuficiência cardíaca. A FM induzida por doenças cardiovasculares é principalmente reparadora, manifestando-se como a transformação de fibroblastos cardíacos em miofibroblastos sob estímulos patológicos quando as células cardíacas são lesionadas. Esses miofibroblastos podem secretar uma grande quantidade de MEC, promovendo a formação de tecido cicatricial, aumentando assim a rigidez cardíaca, levando à FM e resultando em remodelação cardíaca e insuficiência cardíaca. Em contraste, o processo de remodelação da MEC requer a deposição de grandes quantidades de colágeno e outras proteínas; o tipo de colágeno e a reticulação de outroAs proteínas também estão inextricavelmente ligadas à remodelação.^22^ Existem colágenos tipo I e tipo III na MEC. O colágeno tipo I compõe 85% do colágeno total e está principalmente associado às fibras grossas que fornecem resistência à tração, alongamento e deformabilidade. Em contraste, o colágeno tipo III compõe 11% do colágeno total e está associado às fibras finas que fornecem elasticidade, e a deposição de colágeno de ambos os tipos pode diminuir a complacência miocárdica.^23^ Atualmente, o tratamento clínico da FM emprega principalmente intervenções farmacológicas visando o sistema renina-angiotensina-aldosterona para desacelerar a progressão da fibrose; no entanto, os resultados têm sido subótimos.^24^ O transplante cardíaco pode prolongar a sobrevivência do paciente, mas traz consigo seu próprio conjunto de desafios, como riscos elevados, complicações múltiplas e suscetibilidade à rejeição do enxerto, que afetam negativamente os resultados do paciente.^25^ Consequentemente, compreender os mecanismos subjacentes da FM, identificar alvos de tratamento viáveis, inibir a proliferação de fibroblastos cardíacos e atenuar a progressão da fibrose são de vital importância no tratamento de distúrbios cardiovasculares. Este estudo teve como objetivo identificar a FM em um estágio inicial e encontrar marcadores biológicos com alta sensibilidade e especificidade para o desenvolvimento de novas estratégias de tratamento. Nesta pesquisa, GDE na FM foram obtidos dos conjuntos de dados GSE123018, GSE152250 e GSE225336, construindo uma rede PPI compreendendo 37 nós e 71 arestas. Alvos principais foram identificados por meio da rede, e um potencial cluster funcional foi descoberto usando o plugin MCODE.

Por meio da análise de enriquecimento GO e KEGG, os genes-chave foram enriquecidos principalmente em vias intimamente relacionadas à fibrose, como a via de sinalização TGF-β, interação MEC-receptor, cardiomiopatia dilatada e cardiomiopatia hipertrófica. Além disso, esses genes estão envolvidos em processos biológicos, como a interação citocina-receptor citocina, a via de sinalização p53, a via de sinalização Hippo e migração mesenquimal, enfatizando os papéis centrais da apoptose, colágeno, MEC e adesão celular na fibrose. A via de sinalização TGF-β foi amplamente confirmada como tendo um papel regulador significativo na FM.^26^ A expressão elevada de TGF-β é quase universalmente observada em pacientes com FM.^27-29^ Essa expressão diferencial leva à ativação de fibroblastos, estimula a expressão de inibidores teciduais de metaloproteinases da matriz e inibe a atividade das metaloproteinases da matriz, resultando em aumento da deposição de MEC e exacerbação da FM.^30,31^ Numerosos estudos indicam que a FM pode ser significativamente melhorada pela inibição da via de sinalização do TGF-β por meio de medicamentos.^32-34^ Ambas as vias de sinalização P53 e Hippo medeiam a apoptose celular.

Estudos demonstraram que a inibição dessas vias pode suprimir a apoptose dos cardiomiócitos e reduzir a formação de FM.^35,36^ Além disso, a pesquisa indica que o SO2 pode inibir a via Hippo-MST, aliviando a apoptose celular e o estresse do retículo endoplasmático, atenuando significativamente a FM em ratos diabéticos.^37^ Da mesma forma, a análise de enriquecimento do módulo central revelou que suas funções estão intimamente relacionadas aos principais mecanismos da FM, como a montagem da MEC e a migração mesenquimal. Como um suplemento à análise de enriquecimento do gene central, a GSEA continua a destacar a deposição da MEC como um processo central na FM. Além disso, ela introduz as vias inflamatórias de sinalização Hedgehog, mTORC1 e TNF-α por meio do NF-κB, que foram todas confirmadas como relacionadas à FM em vários estudos globais.^38-40^

A expressão gênica em organismos biológicos depende da regulação de miRNAs. Após prever miRNAs para genes hub, foi descoberto que miRNA-148a-3p, miRNA-148b-3p, miR-152-3p, miR-372-3p, miR-506-3p, a família miR-130, a família miR-302, a família miR-29 e a família miR-520 são compartilhadas pela maioria dos genes. A família miR-29, em particular, é uma das famílias de miRNA mais amplamente estudadas na FM. Numerosos estudos mostraram que a família miR-29 pode atingir uma variedade de genes relacionados à síntese de MEC, como várias proteínas de colágeno. Sua regulação negativa na fibrose pode levar à deposição excessiva de componentes da MEC.^41,42^ Em um experimento animal, Sheng-song Xu et al. descobriram que a expressão de miR-152-3p reduzia a proliferação de fibroblastos cardíacos (FC) e aliviava a FM ao inibir a via de sinalização Wnt1/β-catenina.^43^

Da mesma forma, a regulação negativa do miR-130 promove efeitos cardioprotetores mediados pelo PPAR- γ por meio da inibição da inflamação e da FM.^44^ Portanto, a família hsa-miR-29, miR-152-3p e miR-130 poderiam potencialmente servir como marcadores biológicos e alvos terapêuticos para FM. A análise de fatores de transcrição indicou que JUN, NF-κB1, SP1, RELA, SRF e STAT3 estão envolvidos em vias relacionadas à inflamação, proliferação celular, apoptose e respostas ao estresse celular. NF-κB1 e RELA são parte do complexo NF-Κb.^45^ Geng-Rui Xu descobriu que, ao inibir a via de sinalização TLR4/MyD88/NF-κB, a deposição de colágeno foi significativamente reduzida, melhorando assim a FM.^46^ STAT3 está intimamente relacionado à FM e está envolvido em múltiplas vias de sinalização. Estudos demonstraram que a inibição das vias IL-6/STAT3 e JAK-STAT3 alivia o grau de FM.^47,48^

Após realizar análise de especificidade e sensibilidade usando ROC, identificamos genes com AUC maior que 0,9, incluindo IL11, GADD45B, GDF5, NOX4, IGFBP3, ACTC1, MYOZ2 e ITGB8. IL11 é um gene profibrótico clássico. Experimentos recentes mostraram que a secreção de IL11 pode promover sinalização parácrina em fibroblastos adjacentes, bem como transição epitelial-mesenquimal e transição endotelial-mesenquimal, exibindo efeitos profibróticos e pró-inflamatórios evidentes.^49,50^ Em outro estudo, foi descoberto que a IL-11 pode sinergizar ou aumentar os efeitos fibróticos do TGF-β.^51^ Tongtong Song et al. descobriram em experimentos celulares que a inibição de IL11 em células reduziu significativamente a expressão de mRNA e proteína do colágeno tipo I e colágeno tipo III, melhorando assim a remodelação da MEC.^52^ GADD45B é um gene que é regulado positivamente em danos ao DNA e respostas ao estresse, desempenhando papéis em vários processos biológicos, particularmente na regulação do ciclo celular, apoptose, reparo do DNA e respostas ao estresse celular.^53^ A pesquisa mostrou que a driamicina aumentou significativamente o colágeno tipo I, e o colágeno tipo III, diminuiu a função cardíaca, e induziu FM na MEC pela regulação positiva de GADD45B e a ativação de p38 e pJNK no coração.^54^ GDF5 pode regular a expressão de p38-MAPK, subsequentemente inibindo a transcrição de colágeno tipo I e Col1a1 e colágeno tipo III, bem como genes Col3a1 em células cardíacas. Isso leva a uma redução na FM após infarto do miocárdio.^55^ NOX4 é um membro da família NADPH oxidase que medeia a produção de ROS. Numerosos estudos demonstraram que a inibição do estresse oxidativo induzido por NOX4 pode ter efeitos antifibróticos no miocárdio.^56^ Além disso, o NOX4 é regulado pelo TGF-β, uma via fundamental na FM.^57^ Além disso, em um experimento in vivo e in vitro, Nox4 foi encontrado mediando no meio da via TGFβ1 para promover a síntese de colágeno, mas, infelizmente, o experimento não elucidou qual colágeno especificamente.^58^ Da mesma forma, experimentos recentes mostraram que IGFBP3 é altamente expresso em fibroblastos em modelos murinos de FM.^59^ Li et al. descobriram que a inibição da expressão de IGFBP3 atenuou a expressão de colágeno I e III na FM induzida por diabetes.^60^ Um estudo de Alessandra Ruggiero descobriu que camundongos mutantes MYOZ2 podem desenvolver hipertrofia de células cardíacas e fibrose intersticial.^61^ ACTC1 é uma proteína-chave nos miócitos cardíacos e está envolvida em sua função contrátil. Embora mutações em ACTC1 sejam conhecidas por estarem associadas a certas doenças cardíacas, como a cardiomiopatia hipertrófica familiar (HCM),^62^ atualmente não há evidências definitivas que liguem ACTC1 diretamente à FM. Mais pesquisas são necessárias para elucidar essa relação. ITGB8 é um membro da família integrina e está envolvido em interações entre células, bem como entre células e a MEC.^63^ Estudo indica que ITGB8 participa principalmente do processo de fibrose pulmonar, mas seu papel na FM ainda não foi totalmente estabelecido.^64^ No entanto, outros membros da família das integrinas, como o ITGB1, são conhecidos por estarem associados à FM,^65^ garantindo uma investigação mais aprofundada sobre as funções do ITGB8.

**Figura 8:**
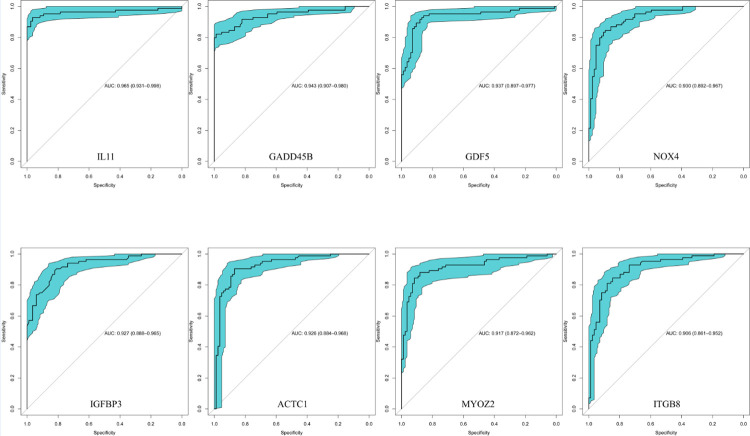
– ROCs de genes centrais.

Este estudo tem certas limitações, pois os resultados que obtivemos foram derivados de conjuntos de dados do banco de dados GEO. Embora esforços tenham sido feitos para controlar e normalizar os efeitos em três conjuntos de dados experimentais, as descobertas não foram verificadas em experimentos do mundo real. Além disso, ao adquirir os conjuntos de dados originais, por uma questão de consistência entre os três conjuntos de dados, optamos pelo grupo experimental induzido por TGF-β de 24 horas sem selecionar os grupos experimentais de 48 ou 72 horas. Embora uma literatura substancial sugira que fibrose significativa pode ser observada após 24 horas de indução, essa abordagem pode ter perdido informações cruciais no processo dinâmico. Além disso, o biomarcador central da FM, TGF-β, não exibiu altos níveis de confiança no conjunto de dados GSE97358, apesar de sua AUC ser 0,882015306. Isso requer uma experimentação extensiva para verificar a especificidade e a sensibilidade desses biomarcadores potenciais.

## Conclusão

Os oito genes, IL11, GADD45B, GDF5, NOX4, IGFBP3, ACTC1, MYOZ2 e ITGB8, podem servir como biomarcadores candidatos para FM. Processos como apoptose celular, síntese de proteína de colágeno, formação de MEC, adesão celular e inflamação estão implicados no desenvolvimento da FM.
