# Supplementary material for: Biomarcadores Potenciais na Fibrose Miocárdica: Uma Análise Bioinformática
Source: Arq Bras Cardiol. 2024 Nov 22;121(12):e20230674. [Article in Portuguese] doi: 10.36660/abc.20230674 (PMC11634303; doi:10.36660/abc.20230674)
Supplement: Translation

# Translation: Potential Biomarkers in Myocardial Fibrosis: A Bioinformatic Analysis

**DOI:** 10.36660/abc.20230674i

**Authors:** Wang Cheng-Mei, Gang Luo, Ping Liu, Wei Ren, Sijin Yang

**Affiliations:** 1 China Beibei Traditional Chinese Medicine Hospital, Chongqing - China; 2 China The Affiliated Hospital of Traditional Chinese Medicine of Southwest Medical University, Luzhou, Sichuan – China

**Keywords:** Fibrosis Endomiocárdica, Biomarkers, MicroRNAs

## Abstract

**Background:**

Myocardial fibrosis (MF) occurs throughout the onset and progression of cardiovascular disease, and early diagnosis of MF is beneficial for improving cardiac function, but there is a lack of research on early biomarkers of MF.

**Objectives:**

Utilizing bioinformatics techniques, we identified potential biomarkers for MF.

**Methods:**

Datasets related to MF were sourced from the GEO database. After processing the data, differentially expressed genes were screened. Differentially expressed genes were enriched, and subsequently, protein-protein interaction (PPI) was performed to analyze the differential genes. The associated miRNAs and transcription factors were predicted for these core genes. Finally, ROC validation was performed on the core genes to determine their specificity and sensitivity as potential biomarkers. The level of significance adopted was 5% (p < 0.05).

**Results:**

A total of 91 differentially expressed genes were identified, and PPI analysis yielded 31 central genes. Enrichment analysis showed that apoptosis, collagen, extracellular matrix, cell adhesion, and inflammation were involved in MF. One hundred and forty-two potential miRNAs were identified. the transcription factors JUN, NF-κB1, SP1, RELA, serum response factor (SRF), and STAT3 were enriched in most of the core targets. Ultimately, IL11, GADD45B, GDF5, NOX4, IGFBP3, ACTC1, MYOZ2, and ITGB8 had higher diagnostic accuracy and sensitivity in predicting MF based on ROC curve analysis.

**Conclusion:**

Eight genes, IL11, GADD45B, GDF5, NOX4, IGFBP3, ACTC1, MYOZ2, and ITGB8, can serve as candidate biomarkers for MF. Processes such as cellular apoptosis, collagen protein synthesis, extracellular matrix formation, cellular adhesion, and inflammation are implicated in the development of MF.

## Introduction

Cardiovascular diseases are common, and their mortality rate surpasses that of other systemic illnesses.^1^ They claim millions of lives annually, constituting approximately 25% of deaths worldwide.^2^ Myocardial fibrosis (MF) plays a pivotal role in the onset and progression of conditions such as atrial fibrillation, coronary artery disease, myocardial infarction, dilated cardiomyopathy, and hypertrophic cardiomyopathy.^3-6^ MF is caused by the abnormal deposition of fibrous tissues in the myocardium. It is characterized by an excessive accumulation of the extracellular matrix (ECM) and a significant increase in collagen, leading to cardiac stiffness and a decline in heart function, followed by electrical remodeling, arrhythmias, and heart failure.^7^

From a pathophysiological standpoint, MF is intricately tied to the reparative mechanisms after cardiac damage. Cardiomyocytes are non-regenerative cells. When these cells are damaged or die, surrounding cells release a series of signaling molecules, such as neurohumoral mediators, cytokines, and growth factors, which induce fibroblast activation to enter the damaged area and produce large amounts of collagen.^8^ Collagen is a primary component of tissue structure, and its excessive accumulation can lead to abnormal deposition of the extracellular matrix, thereby reducing cardiac compliance and impairing its function.^9^ As fibrosis progresses, the heart’s systolic and diastolic functions are further compromised, eventually resulting in heart failure and significantly affecting patients’ quality of life.

Many cardiovascular diseases can manifest as MF at an early stage.^10^ With the global aging population and the rise of lifestyle-related diseases such as atrial fibrillation, hypertension, coronary heart disease, and diabetes, the incidence of MF is steadily increasing, posing a severe threat to human health. Regrettably, current treatments mainly focus on slowing the progression of fibrosis but cannot effectively reverse it.^11^ This is because cardiomyocytes cannot adequately replace dead cells, and the fibrosis process involves extremely complex cells and signaling pathways. Therefore, with the technological advancements in gene chips, sequencing techniques, and whole-genome sequencing, early identification of MF and searching for highly sensitive and specific biomarkers to devise new therapeutic strategies to reverse this condition becomes paramount.

Originating from a bioinformatics perspective, the study identified and analyzed hub genes differentially expressed in MF, investigating their prospects as biomarkers. The core processes of this study can be viewed in the Central Figure.

## Methods

### Obtaining a GeneChip

The Gene Expression Omnibus^12^ (GEO) was used to screen three microarray datasets related to MF and their gene annotation files, specifically GSE 123018/GPL11154, GSE152250/GPL20301, and GSE225336/GPL24676. The GSE123018 raw data contained data from four individuals; GSE152250 and GSE225336 used data from three individuals. All three sets of data contained cardiomyocytes induced for 24 hours using TGFβ1, so we chose the data set processed at 24 hours, and in order to make the data less differentiated, we randomly picked the cell data from the three patients in GSE123018. All these data are open-source and thus do not require review by an ethics committee.

### Screening for differentially expressed genes

Gene chip-related files were sourced and downloaded from the GEO database. This research utilized datasets from three distinct origins, leading to variances in the laboratory handling of each gene chip. Consequently, the removal of batch effects and data normalization became paramount.^13^ The sva package in R (Version 4.2.1) was initially applied for batch effect estimation, followed by the employment of the combat function for its mitigation. Linear models and Bayesian methods in the limma package were used to evaluate gene expression levels under different experimental conditions, correct the variance estimates of individual genes, and normalize the gene matrices using the normalize BetweenArrays function, respectively. In order to select genes with significant differential expression, we set a threshold of |log2-fold change| value more than 2 and P-value less than 0.05 on the selection criteria. Visualization techniques were implemented with the ggplot2 and pheatmap packages.

### Analysis of protein-protein interactions

Protein-protein interaction (PPI)^14^ analysis offers insights into which other proteins a specific protein interacts with within an organism. This aids in better characterizing the functions of these proteins and can shed light on disease onset and progression. Differentially expressed genes (DEGs) were input into the STRING database (https://stringdb.org),^15^ which is an online analysis tool that includes data integration scoring, probabilistic modeling, hypergeometric testing, network topology analysis, and false discovery rate (FDR) correction, and the TSV file of the PPI network was exported. This file was subsequently loaded into Cytoscape (version 3.8.2)^16^ for visualization. The CytoNCA plugin calculates target metrics such as degree, closeness, and betweenness, allowing for the selection of core targets. The MCODE plugin is then utilized to identify potential biomolecular complexes.

### Gene enrichment analysis

Utilizing Metascape (https://Metascape.org/),^17^ a web platform for functional enrichment analysis of large gene sets, Gene Ontology (GO) and Kyoto Encyclopedia of Genes and Genomes (KEGG) analyses of DEGs and potential biomolecular complexes were performed using hypergeometric tests, Benjamini-Hochberg method multiple test correction method. Entries with a p value <0.01 were deemed significantly enriched, and the top 15 results from the GO enrichment analysis and KEGG pathways were selected.

### Gene set enrichment analysis

To comprehensively enhance the understanding of gene functions, GSEA software (version 4.3.2)^18^ was employed on the entire dataset for functional enrichment analysis, encompassing GO, KEGG pathways, and Reactome analysis. For determining significant enrichment, criteria were established as follows: a normalized enrichment score exceeding 1.5, a nominal p-value less than 0.05, and a FDR below 0.25.

### Acquisition of critical miRNAs and transcription factors

The Hub genes were analyzed for potential miRNA using FunRich software (version 3.1.3),^19^ and transcription factor prediction was carried out using TRRUST version 2 (https://www.grnpedia.org/trrust/).^20^

### Gene set validation based on the receiver operating characteristic curve (ROC)

To validate the robustness of the identified hub genes and to avoid overfitting the original data, it is crucial to conduct ROC^21^ validation on an independent dataset to evaluate the hub genes as potential biomarkers. We utilized the gene microarray dataset GSE97358, which includes 84 normal samples and 84 fibrotic samples. ROC analysis and visualization were conducted in R software using the pROC package.

## Results

### Analysis of DEGs

After the original dataset was batch-corrected and normalized, a differential analysis was conducted using the limma package. Ultimately, 91 DEGs were identified, with 56 genes significantly upregulated and 35 genes significantly downregulated. Gene heatmaps and volcano plots of DEGs are illustrated in Figure 1. The specific topological parameters of the DEGs can be found in Table S1.

**Figure 1:** – A) Gene volcano plot. B) Gene heat map.

### PPI analysis

The 91 DEGs were uploaded to the STRING website for PPI analysis. The analysis results were imported into Cytoscape for further investigation. Figure 2A depicts the network consisting of 37 nodes and 71 edges. Gene degrees were calculated using CytoNCA, and by sorting values greater than the median,28 hub genes were identified. The specific topological parameters of the hub genes can be found in Table S2. Meanwhile, the MCODE plugin revealed a potential protein complex functional module (Figure 2B).

**Figure 2:** – A) PPI network. B) bio-functional modules.

### Functional enrichment

Differential gene expression analysis highlighted that, as shown in Figure 3A, the genes participate in biological processes such as mesenchymal migration, connective tissue development, myocardial tissue development, response to mechanical stimulus, circulatory system process, and striated muscle tissue development. Figure 3B demonstrates involvement in various molecular biological functions, such as cell adhesion molecule binding, integrin binding, growth factor activity, glycosaminoglycan binding, collagen binding, extracellular matrix structural constituents, and oxidoreductase activity. The cellular components implicated include the extracellular matrix, external encapsulating structures, collagen-containing extracellular matrix, secretory granule lumen, cytoplasmic vesicle lumen, and myofibrils, as displayed in Figure 3C. KEGG pathway analysis indicated involvement in multiple signaling pathways, such as Hippo signaling pathway, Cytokine-cytokine receptor interaction, Hypertrophic cardiomyopathy, ECM-receptor interaction, Arrhythmogenic right ventricular cardiomyopathy, Cellular senescence, transforming growth factor-β(TGF-β)signaling pathway, Dilated cardiomyopathy, and p53 signaling pathway, which can be observed in Figure 3D, All enrichment analyses with p-values less than 0.05 are shown in Table S3. Enrichment analysis of the biological module showed involvement in extracellular matrix assembly and mesenchymal migration, as shown in Figure 4.

**Figure 3:** – A) GO biological processes. B) GO molecular function. C) GO cellular components. D) KEGG.

**Figure 4:** – Enrichment analysis of the biological module.

### GSEA results

Upon conducting GSEA on the fully adjusted gene dataset, Reactome revealed primary enrichments in TGF-β, Hallmark tumor necrosis factor-α(TNF-α) signaling via NF-κB, mTorc1 signaling, Hedgehog signaling, and unfolded protein response. The main cellular components included the subunit processome, pre-ribosome, and complex collagen trimers. In terms of biological functions, gene participation was observed in transcription regulator inhibitor activity, extracellular matrix structural constituent, and growth factor activity. KEGG pathway analysis showed involvement in the TGF-β signaling pathway, glycosaminoglycan biosynthesis, chondroitin sulfate, and dilated cardiomyopathy. Regrettably, in GO biological processes, no gene sets were significant at an FDR below 25%, as depicted in Figure 5, and the relevant parameters can be found in Table S4.

**Figure 5:** – GSEA.

### Prediction of miRNAs and transcription factors

Utilizing FunRich to predict miRNAs, abundance analysis retained miRNA-148a-3p, miRNA-148b-3p, miR-152-3p, miR-372-3p, miR-506-3p, the miR-130 family, the miR-302 family, the miR-29 family, and the miR-520 family, as displayed in Figure 6. Postanalysis prediction of transcription factors through the TRRUST platform revealed that Jun proto-oncogene (JUN), NF-κB1, Sp1 transcription factor (SP1), RELA proto-oncogene (RELA), SRF, and signal transducer and activator of transcription 3 (STAT3) were highly enriched among the majority of core targets, as illustrated in Figure 7.

**Figure 6:** – miRNAs.

**Figure 7:** – Transcription factors analysis.

### The results of the ROC validation

During the ROC analysis of core targets, the study found that the AUCs for the genes interleukin 11(IL11), growth arrest and DNA damage-inducible beta (GADD45B), growth differentiation factor 5 (GDF5), NADPH oxidase 4 (NOX4), insulin-like growth factor binding protein 3 (IGFBP3,) actin alpha cardiac muscle 1(ACTC1), myozenin 2 (MYOZ2), and integrin subunit beta 8 (ITGB8) were 0.964710884, 0.943239796, 0.936791383, 0.929563492, 0.92651644, 0.926232993, 0.91723356, and 0.906391723, respectively. All AUCs were greater than 0.9; among them, IL11, GADD45B, NOX4, IGFBP3, and ACTC1 are upregulated in fibrosis, while GDF5, MYOZ2, and ITGB8 are downregulated in expression, substantiating these genes as potential biological markers for diagnosing MF.

## Discussion

Cardiovascular diseases, as the leading cause of death worldwide, pose a significant burden on global public health.^1^ Common cardiovascular conditions, such as coronary heart disease, myocardial infarction, hypertensive heart disease, cardiomyopathy, atrial fibrillation, and diabetic cardiomyopathy, can all lead to MF, ultimately resulting in heart failure. MF induced by cardiovascular diseases is primarily reparative, manifesting as the transformation of cardiac fibroblasts into myofibroblasts under pathological stimuli when cardiac cells are injured. These myofibroblasts can secrete a large amount of extracellular matrix, promoting scar tissue formation, thereby increasing cardiac stiffness, leading to MF, and resulting in cardiac remodeling and heart failure. In contrast, the remodeling process of the ECM requires the deposition of large amounts of collagen and other proteins; the type of collagen and cross-linking of other proteins are also inextricably linked to remodeling.^22^ There are type I and type III collagens in ECM. Type I collagen makes up 85% of the total collagen and is primarily associated with the coarse fibers that provide tensile strength, stretch, and deformability. In contrast, type III collagen makes up 11% of the total collagen and is associated with the fine fibers that provide elasticity, and collagen deposition of both types can decrease myocardial compliance.^23^ At present, the clinical management of MF mainly employs pharmacological interventions targeting the renin-angiotensin-aldosterone system to decelerate fibrosis progression; however, the results have been suboptimal.^24^ Cardiac transplantation can prolong patient survival but comes with its own set of challenges, such as elevated risks, multiple complications, and susceptibility to graft rejection, which adversely affect patient outcomes.^25^ Consequently, understanding the underlying mechanisms of MF, pinpointing viable treatment targets, inhibiting cardiac fibroblast proliferation, and attenuating the progression of fibrosis are of vital importance in the management of cardiovascular disorders. This study aimed to identify MF at an early stage and to find biological markers with high sensitivity and specificity for the development of new treatment strategies. In this research, DEGs in MF were obtained from the GSE123018, GSE152250, and GSE225336 datasets, constructing a PPI network comprising 37 nodes and 71 edges. Core targets were identified through the network, and a potential functional cluster was discovered using the MCODE plugin.

Through GO and KEGG enrichment analysis, the key genes were mainly enriched in pathways closely related to fibrosis, such as the TGF-β signaling pathway, ECM-receptor interaction, dilated cardiomyopathy, and hypertrophic cardiomyopathy. In addition, these genes are involved in biological processes such as cytokine‒cytokine receptor interaction, the p53 signaling pathway, the Hippo signaling pathway, and mesenchymal migration, emphasizing the central roles of apoptosis, collagen, extracellular matrix, and cell adhesion in fibrosis. The TGF-β signaling pathway has been widely confirmed to play a significant regulatory role in MF.^26^ Elevated expression of TGF-β is almost universally observed in patients with MF.^27-29^ This differential expression leads to the activation of fibroblasts, stimulates the expression of tissue inhibitors of matrix metalloproteinases, and inhibits the activity of matrix metalloproteinases, resulting in increased deposition of extracellular matrix and exacerbation of MF.^30,31^ Numerous studies indicate that MF can be significantly improved by inhibiting the TGF-β signaling pathway through medication.^32-34^ Both the P53 and Hippo signaling pathways mediate cell apoptosis.

Studies have shown that inhibiting these pathways can suppress cardiomyocyte apoptosis and reduce the formation of MF.^35,36^ Additionally, research indicates that SO2 can inhibit the Hippo-MST pathway, alleviating cell apoptosis and endoplasmic reticulum stress (ERS), thus significantly attenuating MF in diabetic rats.^37^ Similarly, enrichment analysis of the core module revealed that its functions are closely related to key mechanisms of MF, such as extracellular matrix assembly and mesenchymal migration. As a supplement to the core gene enrichment analysis, GSEA continues to highlight the deposition of the extracellular matrix as a central process in MF. Additionally, it introduces the Hedgehog signaling, mTORC1, and TNF-α inflammatory pathways through NF-κB, which have all been confirmed to be related to MF in various global studies.^38-40^

Gene expression in biological organisms relies on the regulation of miRNAs. After predicting miRNAs for hub genes, it was found that miRNA-148a-3p, miRNA-148b-3p, miR-152-3p, miR-372-3p, miR-506-3p, the miR-130 family, the miR-302 family, the miR-29 family, and the miR-520 family are shared by a majority of genes. The miR-29 family, in particular, is one of the most extensively studied miRNA families in MF. Numerous studies have shown that the miR-29 family can target a variety of genes related to ECM synthesis, such as various collagen proteins. Its downregulation in fibrosis can lead to excessive deposition of ECM components.^41,42^ In an animal experiment, Sheng-song Xu et al. discovered that the expression of miR-152-3p was found to reduce cardiac fibroblast (CF) proliferation and alleviate MF by inhibiting the Wnt1/β-catenin signaling pathway.^43^

Similarly, the downregulation of miR-130 promotes cardioprotective effects mediated by PPAR-γ through the inhibition of inflammation and MF.^44^ Therefore, the hsa-miR-29 family, miR-152-3p, and miR-130 could potentially serve as biological markers and therapeutic targets for MF. Analysis of transcription factors indicated that JUN, NF-κB1, SP1, RELA, SRF, and STAT3 are involved in pathways related to inflammation, cell proliferation, apoptosis, and cellular stress responses. NF-κB1 and RELA are part of the NF-κB complex.^45^ Geng-Rui Xu found that by inhibiting the TLR4/MyD88/NF-κB signaling pathway, collagen deposition was significantly reduced, thereby ameliorating MF.^46^ STAT3 is closely related to MF and is involved in multiple signaling pathways. Studies have shown that inhibiting the IL-6/STAT3 and JAK-STAT3 pathways alleviates the degree of MF.^47,48^

After performing specificity and sensitivity analysis using ROC, we identified genes with an AUC greater than 0.9, including IL11, GADD45B, GDF5, NOX4, IGFBP3, ACTC1, MYOZ2, and ITGB8. IL11 is a classic profibrotic gene. Recent experiments have shown that IL11 secretion can promote paracrine signaling in adjacent fibroblasts, as well as epithelial-to-mesenchymal transition (EMT) and endothelial-to-mesenchymal transition (EndMT), displaying evident profibrotic and proinflammatory effects.^49,50^ In another study, it was found that IL-11 can synergize with or enhance the fibrotic effects of TGF-β.^51^ Tongtong Song et al. found in cellular experiments that inhibition of IL11 in cells significantly reduced mRNA and protein expression of type I collagen and type III collagen, thereby improving ECM remodeling.^52^ GADD45B is a gene that is upregulated in DNA damage and stress responses, playing roles in various biological processes, particularly in cell cycle regulation, apoptosis, DNA repair, and cellular stress responses.^53^ Research has shown that adriamycin significantly increased type I collagen, and type III collagen, decreased cardiac function, and induced MF in the ECM by upregulating GADD45B and activating p38 and pJNK in the heart.^54^ GDF5 can regulate the expression of p38-MAPK, subsequently inhibiting the transcription of type I collagen and Col1a1 and type III collagen, as well as Col3a1 genes in cardiac cells. This leads to a reduction in MF following myocardial infarction.^55^ NOX4 is a member of the NADPH oxidase family that mediates the production of ROS. Numerous studies have shown that inhibiting NOX4-induced oxidative stress can have antifibrotic effects on the myocardium.^56^ Additionally, NOX4 is regulated by TGF-β, a fundamental pathway in MF.^57^ Furthermore, in an in vivo and in vitro experiment, Nox4 was found mediating in the middle of the TGFβ1 pathway to promote collagen synthesis, but, unfortunately, the experiment did not elucidate which collagen specifically.^58^ Likewise, recent experiments have shown that IGFBP3 is highly expressed in fibroblasts in mouse models of MF.^59^ Changlin Li et al. found that inhibition of IGFBP3 expression attenuated collagen I and III expression in diabetes-induced MF.^60^ A study by Alessandra Ruggiero found that MYOZ2 mutant mice can develop cardiac cell hypertrophy and interstitial fibrosis.^61^ ACTC1 is a key protein in cardiac myocytes and is involved in their contractile function. Although mutations in ACTC1 are known to be associated with certain cardiac diseases, such as familial hypertrophic cardiomyopathy (HCM),^62^ there is currently no definitive evidence linking ACTC1 directly to MF. Further research is needed to elucidate this relationship. ITGB8 is a member of the integrin family and is involved in interactions between cells as well as between cells and the ECM.^63^ Study indicates that ITGB8 primarily participates in the process of pulmonary fibrosis, but its role in MF has not yet been fully established.^64^ However, other members of the integrin family, such as ITGB1, are known to be associated with MF,^65^ warranting further investigation into the functions of ITGB8.

This study has certain limitations, as the results we obtained were derived from GEO database datasets. While efforts have been made to control and normalize the effects in three experimental datasets, the findings have not been verified in real-world experiments. Furthermore, in acquiring the original datasets, for the sake of consistency across the three datasets, we opted for the 24-hour TGF-β-induced experimental group without selecting the 48-hour or 72-hour experimental groups. Although substantial literature suggests that significant fibrosis can be observed after 24 hours of induction, this approach might have missed crucial information in the dynamic process. Moreover, the core biomarker of MF, TGF-β, did not exhibit high confidence levels in the GSE97358 dataset despite its AUC being 0.882015306. This necessitates extensive experimentation to verify the specificity and sensitivity of these potential biomarkers.

**Figure 8:** – ROCs of hub genes.

## Conclusion

The eight genes, IL11, GADD45B, GDF5, NOX4, IGFBP3, ACTC1, MYOZ2, and ITGB8, can serve as candidate biomarkers for MF. Processes such as cellular apoptosis, collagen protein synthesis, extracellular matrix formation, cellular adhesion, and inflammation are implicated in the development of MF.
